# Long-Term Consequences of Developmental Alcohol Exposure on Brain Structure and Function: Therapeutic Benefits of Physical Activity

**DOI:** 10.3390/brainsci3010001

**Published:** 2012-12-21

**Authors:** Anna Y. Klintsova, Gillian F. Hamilton, Karen E. Boschen

**Affiliations:** Department of Psychology, University of Delaware, Newark, DE 19716, USA; E-Mails: gillianh@psych.udel.edu (G.F.H.); kboschen@psych.udel.edu (K.E.B.)

**Keywords:** fetal alcohol, adolescence, animal model, plasticity, hippocampus, prefrontal cortex, apoptosis, neurotrophins

## Abstract

Developmental alcohol exposure both early in life and during adolescence can have a devastating impact on normal brain structure and functioning, leading to behavioral and cognitive impairments that persist throughout the lifespan. This review discusses human work as well as animal models used to investigate the effect of alcohol exposure at various time points during development, as well as specific behavioral and neuroanatomical deficits caused by alcohol exposure. Further, cellular and molecular mediators contributing to these alcohol-induced changes are examined, such as neurotrophic factors and apoptotic markers. Next, this review seeks to support the use of aerobic exercise as a potential therapeutic intervention for alcohol-related impairments. To date, few interventions, behavioral or pharmacological, have been proven effective in mitigating some alcohol-related deficits. Exercise is a simple therapy that can be used across species and also across socioeconomic status. It has a profoundly positive influence on many measures of learning and neuroplasticity; in particular, those measures damaged by alcohol exposure. This review discusses current evidence that exercise may mitigate damage caused by developmental alcohol exposure and is a promising therapeutic target for future research and intervention strategies.

## 1. Introduction: Protective Effect of Exercise on the Human Brain

This review focuses on the potential therapeutic effects of exercise on the brain after developmental alcohol exposure. Exercise has been proven valuable because it increases neuronal plasticity, enhances both angiogenesis and neurogenesis, reduces inflammation, and suppresses oxidative stress (reviewed in [1,2]). Successful therapies to rehabilitate the adverse effects of exposure to alcohol during development on a child’s brain do not exist yet. This review will describe findings from studies that examine the promising influence of exercise as a therapeutic intervention in relation to experimental models of fetal and adolescent alcohol exposure. Animal studies have allowed us to obtain reliable biomarkers both for brain damage due to alcohol exposure and for neuroplasticity due to various forms of exercise. First, we will describe existing animal models of exposure to alcohol during different trimesters of human pregnancy and during adolescence. Second, we will explore the mechanisms behind alcohol-induced brain damage and exercise-induced neuroplasticity and neuroprotection. Finally, we will summarize the most recent studies where exercise was used in attempt to ameliorate damage produce by developmental alcohol exposure in animal models.

The effects of exercise on overall health are well known and described in children, adults, and the elderly [3,4,5,6,7,8,9,10]. Physical activity has been shown to promote brain plasticity perhaps by enhancing both structural and functional parameters of the central nervous system (CNS), e.g., cerebral blood flow [6]. Exercise improves learning, reaction time, and processing speed in older people [11]. These improvements have been commonly connected with neuroplasticity in the hippocampus. While physical activity was demonstrated to improve cognitive function (increased ERP amplitude and decreased latency; increased gray matter volume) in the elderly subjects, decreased mobility in elderly is correlated with cognitive decline and higher risk of Alzheimer’s disease [12]. Neuroprotective effects of vigorous exercise and physical fitness, especially when exercise during midlife, are demonstrated by significant decrease of the risk of Parkinson’s disease, dementia and cognitive impairment [13].

The goal of this review is to summarize the most recent findings and reports regarding the effect of exercise (in adolescence, adulthood or old age; in humans and animals) on plasticity in the brain affected by developmental exposure to alcohol. Before we can address any possibility of using exercise to enhance brain plasticity it is necessary to summarize what is known about the consequences of exposure to alcohol *in utero* or at later developmental time points (adolescence) and how basic science utilizes animal models to understand these effects. We then review the recent reports on the importance of exercise in recovery from exposure to alcohol during development (pre- or postnatal, or during adolescence).

## 2. Fetal Alcohol Spectrum Disorders, Adolescent Drinking and Modeling of Developmental Alcohol Effects in Experimental Animals

Developmental alcohol exposure most often refers to either drinking by the pregnant mother or to drinking by an individual during adolescence. In both cases, the person is going through significant developmental changes that can be affected by alcohol exposure. This section will describe the clinical significance of research that examines drinking during these time periods, diagnostic signs and symptoms, and specific neurological damage that occurs at each developmental stage.

### 2.1. Impact of Developmental Alcohol Exposure on Society

Fetal Alcohol Spectrum Disorders (FASD) is a term that encompasses various conditions and diagnoses including Alcohol-Related Neurodevelopmental Disorder, Alcohol-Related Birth Defects, and the most severe manifestation, Fetal Alcohol Syndrome (FAS). Despite increasing public awareness over recent decades as to the harmful nature of prenatal alcohol exposure, FASD still is reported in approximately five in 100 (5%) live births each year in the United States, making it a leading cause of preventable mental retardation in children [14,15,16]. The cost of individual diagnosis and lifetime care for a child affected by FASD can be up to $2 million in the most severe cases, with FASD costing the United States up to $4 billion in 1998 in health care and special services costs [15,17].

The symptoms of FASD can vary greatly between individuals based upon factors that include, but are not limited to, the amount of alcohol the mother ingested, the developmental time window when such exposure(s) occurred, and individual factors, such as alcohol metabolism rates. The most severe form, FAS, is characterized by facial malformations, below average birth height and weight, and significant behavioral and cognitive changes with underlying CNS abnormalities. Children with FAS have a flat philtrum, small eye openings, and a thin upper lip. Overall, there is often a general flattening of the facial features that give children with FAS a very distinct look. In addition, there are gross physical changes to the CNS itself, including reduced brain volume [18,19] and increased cortical thickness [20].

While children with FAS display some of the most severe behavioral and cognitive abnormalities, these changes are part of the diagnostic criteria for all forms of FASD [16]. Memory and attention deficits, as well as difficulties with executive functioning, motor skills, and problem-solving, are common in children with FASD. Additionally, hyperexcitability, difficulty socializing with peers, and other conduct problems are often reported [21,22]. Overall, FASD can be a devastating, costly disease with life-long impacts on both the child as well as the community. 

More recently, research has focused on the impact of adolescent alcohol intake, given the increasingly large number of adolescents imbibing and that the present generation of adolescents consists of more than a quarter of the world’s total population [23]. In 2010 alone, the cost of adolescent drinking on society was $62 million dollars [24], suggesting an important need to address this problem. The prevalence of adolescent drinking can be as high as 51.6% in 18–20 year old. In 2006, about 10.8 million people aged 12–20 reported drinking alcohol in the past month. Of those, 7.2 million were binge drinkers (*i.e.*, consuming five or more drinks on a single occasion at least once in the past 30 days) while 2.4 million were heavy drinkers (*i.e.*, consuming five or more drinks on a single occasion 5 or more times in the past 30 days) [18]. In 2009, 16.2% of alcohol sales were made to underage drinkers and 8% of all treatment admissions made for alcohol abuse were by adolescent drinkers [25]. Further, in 2008, approximately, 180,000 hospital visits by adolescent drinker occurred [16]. Together, these statistics illustrate an alarming cost to society that can be prevented. 

### 2.2. Modeling Developmental Alcohol Exposure in Animals

Multiple factors can affect the type and the severity of deficits observed following developmental alcohol exposure. Specifically, some of the most important factors that contribute to the spectrum of exhibited deficits are (1) the timing of the alcohol exposure during development, (2) the dose of alcohol given as measured by blood alcohol concentration (BAC), and (3) the route of alcohol administration. 

**Figure 1:**
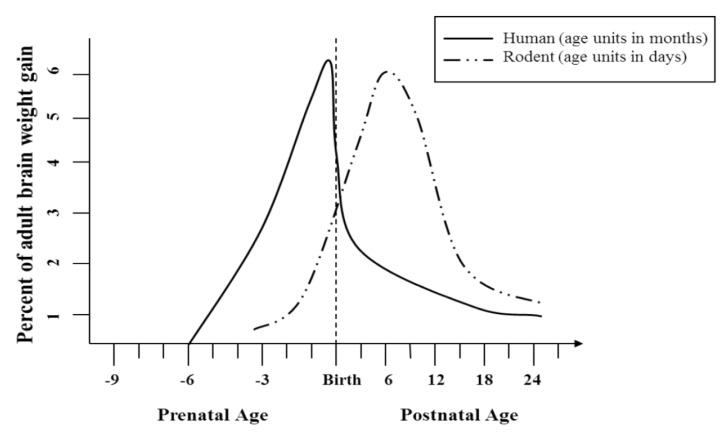
Brain growth spurt in humans and rodents.

Animal models have been very useful in parsing out the effect of timing of alcohol exposure on the type of impairments produced in the individual. Alcohol exposure will most greatly affect structures that are going through a critical period of development during the time of the exposure. For example, alcohol exposure during the first trimester equivalent (gestational days 0–17 in the rodent) can produce the craniofacial malformations observed in children with FAS [26,27]. During the first two weeks after birth in the rodent, which is considered to be an equivalent of the third trimester in human pregnancies [28], a significant amount of neurogenesis and synaptogenesis is occurring in the rodent brain (Figure 1). Alcohol administration during this period, known as “brain growth spurt” causes substantial damage to brain areas completing formation. Regions such as the prefrontal cortex (PFC), hippocampus, and cerebellum are particularly sensitive to alcohol exposure later in fetal development. Exposure during the third trimester equivalent is often used to model less severe forms of FASD that primarily affect cognitive and behavioral measures. The PFC is also vulnerable during adolescent alcohol exposure, particularly in humans since the PFC does not complete development until the mid-twenties [29]. 

Animal models employ various routes of alcohol administration that can affect the severity of damage produced by altering the level and duration of BAC achieved in the animal. The route of administration can be dictated by the developmental window targeted and pattern of exposure being used (ex. prolonged, moderate exposure *vs.* binge-like exposure). The procedures described below are specific to developmental alcohol exposure, including prenatal, neonatal, and adolescent administration. While it is not an exhaustive list, the described approaches are the most commonly used in current alcohol research. Methodological differences between developmental time points will be specified as needed.

Self-administration of alcohol by the dam during gestation has been a common method to model low to moderate patterns of drinking. Alcohol is mixed to the desired concentration with maltose dextrin as a caloric substitute or with sweetened water in place of plain drinking water. A drawback to this model is the inability to exactly control the amount of alcohol intake across animals or, within one animal, across days. In addition, in FASD models, this method can only be used to model alcohol exposure during the equivalents of the first two trimesters. Self-administration is commonly used in adolescent models of drinking.

Another commonly used route of administration is intragastric gavage. This method can be used to target any stage of development either by intubation of the dam during the pregnancy or of the pups during the third trimester equivalent. Gavage has the benefit of being able to produce consistent, high BACs in a physiologically relevant way. This method is particularly useful in mimicking binge-like exposure as the BACs produced can be substantially higher than those achieved through self-administration. However, this method can also be stressful to both the dams and pups, and the contribution of handling stress and changes to maternal care to the deficits seen in the pups have yet to be explored. The method of gavage administration of alcohol to adolescent animals differs slightly than that to pups. Most importantly, the time between individual gavages in adolescent animals is different (less than thirty minutes) in order to obtain a high BAC, while in pups, several hours interval occurs between doses. In addition, a metal tube is used to administer the alcohol in adolescents, while plastic tubing is used for pups. The pattern of alcohol administration is also modified for adolescent animals, as the alcohol is pulsed in delivery for adolescents, while it is a smooth delivery for pups.

Inhalation of alcohol vapors by both the dam and the pups is a more recently established model in which the animals are placed in a chamber for prolonged amounts of time (usually 3–4 h). While this method is not as physiologically relevant as alcohol ingestion, it decreases procedure-related stress, including separation of the pups from the dam. Inhalation also does not allow for exact dose control, but it can achieve higher BACs compared to self-administration. 

Other models of developmental alcohol exposure that less commonly used include intraperitoneal injections and artificial rearing of the pups (“pup in the cup” model). Injections are primarily used for acute alcohol exposure in the pups, as repeated injections can be stressful. However, precise dose can be monitored and a split-litter design is easy to implement with the injection method. Artificial rearing, in which the offspring are implanted with a small tube for alcohol delivery directly into the stomach, has largely fallen out of favor. This change is due to the social isolation stress that this model produces; the pup is reared alone in a cup with bedding floating in a water bath away from both maternal and sibling interaction. Despite its downfalls, this method offered a precise way to deliver repeated alcohol doses to a postnatal pup in a consistent manner.

It is important to understand the method and timing of alcohol dosing when reviewing current literature on animal models of developmental alcohol exposure. However, regardless of the method used, alcohol exposure both *in utero* and in adolescence have been shown to produce profound effects on behavior and neuroanatomical measures. The following section will review current evidence from human studies and animals models regarding the damaging effects of alcohol on the developing brain.

### 2.3. Use of Animal Models in Determining Long-Term Behavioral and Anatomical Effects of Developmental Alcohol Exposure

Developmental alcohol exposure produces various types of behavioral deficits due to the widespread action of alcohol on the brain. A great deal of research has focused on how prenatal, neonatal, and adolescent alcohol exposure affect performance on learning and memory tasks, as well as executive functioning tasks, as these are some of the most common behavioral deficits observed in these age groups. In addition, researchers have investigated gross changes to the brain, such as regional volume and cell number as well as alterations to microstructure (e.g., synapse number, dendritic spine density) and cellular processes (e.g., protein expression and phosphorylation). This section describes relevant recent behavioral and anatomical information separated by developmental exposure time point (FASD models *vs.* adolescent models) and brain region of interest.

#### 2.3.1. Behavioral and Anatomical Effects in Prenatal and Postnatal Models of Developmental Alcohol Exposure

Motor impairments are a common sign of FASD, in part due to alcohol’s damaging effects on the cerebellum, though prenatal alcohol exposure can also affect the basal ganglia [19,30,31,32]. In humans, children with FASD have impaired hand-eye coordination [33] and smaller cerebellar vermes compared to age-matched control children [30,31]. Additionally, in clinical studies of cases of children who died with a diagnosis of FAS, cerebellar abnormalities were reported, possibly due in part to hydrocephaly [34,35]. In rodents, third trimester-equivalent binge-like alcohol exposure resulted in impaired learning on a complex motor task [36]. This behavioral impairment was accompanied by a reduced volume of paramedian lobule in the cerebellum and by a decreased number of parallel fiber synapses on Purkinje cells in alcohol-exposed animals. In addition, neonatal alcohol exposure decreased cerebellar Purkinje cell number independent of exposure method, with exposure on postnatal days (PD) 4–6 being most detrimental (reviewed in [37,38]). More recently, a single neonatal binge decreased Purkinje cell number and induced apoptosis as measured by caspase-3 labeling [39].

Deficits following developmental alcohol exposure have been found on a wide range of hippocampal-associated tasks, including spatial learning in the Morris Water Maze [40,41], trace and contextual fear conditioning [42,43,44,45], and trace eyeblink conditioning in both rodents and human children with FASD [46,47,48,49] (summarized in Table 1). Anatomically, reduced cell number in hippocampus, decreased spine density, altered CA1 long-term potentiation (LTP), impaired survival and maturation of adult-born neurons within the dentate gyrus, and altered hippocampal BDNF levels have been reported after neonatal alcohol exposure [43,50,51,52,53,54] (summarized in Table 2 and Table 3). Overall, the hippocampus has been shown to be very vulnerable to developmental alcohol exposure, particularly during the third trimester-equivalent.

**Table 1:** Effects of developmental alcohol exposure and aerobic exercise on behavioral measures.

Behavioral Measures	Alcohol	Exercise	References	
			Alcohol	Exercise
**Executive functioning**	↓	↑	P: [55,56,57]; A: [58]	[59,60,61]
**Anxiety-like behaviors**	↑	↓	P: [62]; A: [63,64,65]	[62]
**Depression-like behaviors**	↑	↓	P: [62,66]	P: [62,66]
**Alcohol Preference as Adult**	↑	-	A: [67,68]	
**Balance/Fine motor skills**	↓	↑	A: [36,69,70]	[71,72]
**Spatial memory**	↓	↑	P: [40,41]; A: [64,65,73,74]	[75,76,77,78,79]
**Fear conditioning**	↓	↓/↑	P: [42,43,44,45]	[44,80]
**Eyeblink conditioning**	↓	↑	P: [46,47,48,49]	[81,82]
**Normative social behavior**	↓	-	P: [21,33]; A: [83]	
**Long-term potentiation (LTP)**	↓	↑	P: [51,84]	[85]

This table is intended to give a general overview of the current literature regarding the effect of developmental alcohol exposure and exercise, either independently or together, on various behaviors. The references noted here are intended as a partial list of examples. Legend: ↓ = decrease in level or impairment, ↑ = increase in level or enhancement, - = research on this effect has not been done; References: P = Pre/Neonatal models, A = Adolescent models.

**Table 2:** Effects of developmental alcohol exposure and aerobic exercise on molecular measures.

Molecular Measures	Alcohol	Exercise	References	
			Alcohol	Exercise
**BDNF levels**	↓/↑	↑	P: [52,66,86,87,88,89,90,91,92] A: [90]	[75,77,79,93,94,95,96,97]
**VEGF levels**	↑	↑	P: [86]	[98,99]
**Oxidative stress markers**	↑	↓	P: [62,100,101]	P: [62,100,101]
**Apoptotic markers**	↑	↓	P: [39,102,103,104,105,106]	P: [39,102,103,104,105,106]
**Gene methylation**	↑	↓	P: [107]	[108]

This table is intended to give a general overview of the current literature regarding the effect of developmental alcohol exposure and exercise, either independently or together, on cellular protein levels and other molecular measures. The references noted here are intended as a partial list of examples. Legend: ↓ = decrease in level or impairment, ↑ = increase in level or enhancement; References: P = Pre/neonatal models, A = Adolescent models.

**Table 3:** Effects of developmental alcohol exposure and aerobic exercise on neuroanatomical measures.

Neuroanatomical Measures	Alcohol	Exercise	References	
			Alcohol	Exercise
**Regional volume**	↓	↑	P: [ 18,19,30,31,70]	[ 70,76,109]
**Cerebellar cell number**	↓	↑	P: [ 39]	P: [ 39]
**Hippocampal cell number**	↓	↑	P: [ 43]	[ 110]
**Dendritic complexity**	↓	↑	P: [ 111,112]	[113,114]
**Spine density**	↓	↑	P: [ 115]	[ 113]
**Synapse number**	↓	↑	P: [ 36]	P: [ 36]
**Adult neurogenesis**	↓	↑	P: [ 53,54,116,117]; A: [118]	[ 77,98,117,119]
**Microvasculature density**	↓	↑	P: [ 120,121,122,123]	[78,99,124,125,126]

This table is intended to give a general overview of the current literature regarding the effect of developmental alcohol exposure and exercise, either independently or together, on neuroanatomy. The references noted here are intended as a partial list of examples. Legend: ↓ = decrease in level or impairment, ↑ = increase in level or enhancement; References: P = Pre/neonatal models, A = Adolescent models.

Research on alterations to PFC structure and function has been relatively limited compared to other brain structures, particularly in regards to studies of executive function in animal models. Recent research in children has reported executive functioning deficits in those prenatally exposed to alcohol on various scales [55,56]. Additionally, adults with FASD have a 60% likelihood of committing crimes and are an over-represented group in the incarcerated population [57], perhaps due to impairments in judgment and impulse control. In rodent models, analysis of Layer II/III mPFC neurons following developmental alcohol exposure found reduced spine density and dendritic complexity [111,112,115] (Table 3). Recently, neonatal alcohol exposure has been suggested to have epigenetic effects. Otero and colleagues [107] reported increased global methylation of the PFC, which could reduce gene expression and cause downstream behavioral impairments. More research is necessary to further explore the effects of prenatal alcohol exposure on the PFC and how these changes might relate to deficits in executive functioning. 

Prenatal alcohol exposure has also been shown to impact social problem solving in children [21]. When presented with a social dilemma and asked to generate solutions to the problem, children with FASD came up with fewer relevant solutions compared to age-matched controls. In addition, Irner and colleagues [33] found that preschool children who were exposed to alcohol *in utero* had lower scores on a personality and social skill subscale of the Griffiths Mental Development Scales, regardless of environmental factors such as the mother’s social background. Overall, these data suggest that prenatal alcohol exposure can have long-lasting detrimental effects on social intelligence and interpersonal skills.

#### 2.3.2. Behavioral and Anatomical Effects in Adolescent Models of Developmental Alcohol Exposure

Adolescence is a period of immense brain plasticity, which provides the opportunity for important experiences to shape the individual in long-lasting ways. However, it may also render the individual more vulnerable to external influences. In particular, exposure to alcohol during adolescence has been associated with poor outcomes in adulthood, such as increased risk for adult substance dependence and crime [127]. During adolescence, very important neurobiological changes continue to occur due to the maturation processes in the brain, including, but not limited to, developmental changes in the PFC, the cerebellum, and the hippocampus as well as white matter structural alterations [67,84,127,128,129]. Additionally, many neurotransmitter systems, such as GABA and dopamine, continue to develop and mature during adolescence [67,128]. 

The adolescent brain appears to be sensitive to the effects of alcohol, which can in turn influence behavior. In fact, brain regions that undergo the most protracted development during adolescence, such as the PFC and the hippocampus, are strongly implicated as sites where alcohol produces significant detrimental effects [127,128,129]. Repeated alcohol exposure during adolescence produced brain region-specific alterations in NMDA receptor activity. Specifically, significant increases in MK-801 (NMDA antagonist) binding in the frontal cortex was evident while minimum binding of MK-801 was seen in the hippocampus [64]. Further, the expression of the GABA_A_ receptor subunit is strongly influenced by alcohol [128], as is dopamine concentration and fiber density in both the PFC [130] and the nucleus accumbens septi [67]. Binge alcohol exposure during adolescence temporarily inhibited adult hippocampal neurogenesis by specifically targeting the cells in in S-phase of the cell cycle during which the new DNA strands are synthesized [118]. In addition, alcohol significantly increased the number and altered the morphology of newly born microglia in the hippocampus [131]. In the mPFC, adolescent alcohol exposure decreased the number of glial cells in the adult male but not the adult female [132]. Cerebellar Purkinje neurons in adolescent rats failed to exhibit depressed firing rate in response to alcohol, as seen with *in vivo* electrophysiological recordings [84]. Alcohol exposure also impacts glutamatergic transmission [68]. Binge-like exposure to alcohol during adolescence not only alters the dopaminergic system but also causes histone modifications, induces chromatin remodeling, changes histone acetylation and methylation in brain reward regions, and also modifies the effects of alcohol on place conditioning [68]. Together, these data illustrate that alcohol exposure severely impacts the wiring of the developing adolescent brain.

Adolescent alcohol exposure affects performance on hippocampus-associated tasks and social behavior. In humans, binge drinking among college students (18–20 years) is associated with poor verbal declarative memory [58]. Rodent work links adolescent alcohol exposure to deficits in spatial learning, evident through performance on the Morris Water Maze [65,73,74,133] and increased anxiety-related behavior, as measured by the elevated plus maze [63]. Further, adolescent rats exposed to alcohol exhibit an increased social avoidance when sober, a trait not seen in adult rats exposed to alcohol [83] and also exhibit increased preference and intake of alcohol in adulthood [67,68]. Additionally, adolescent rats exposed given restricted access to alcohol drinking water exhibited impaired performance in tone conditioning when compared to adults exposed to the same drinking water [134]. Still, adolescents consume more alcohol than do adults [134] and impairments in behavioral performances of adolescents appear to be much more long lasting following the cessation of alcohol-exposure when compared to adults [64]. Therefore, it appears alcohol exposure during adolescence significantly impairs proper functioning, and, potentially, wiring of the developing brain.

## 3. Mechanisms Underlying Alcohol and Exercise Effects on the Developing Brain

Developmental alcohol exposure has widespread effects on the brain. Alcohol primarily affects neurons as an NMDA antagonist and GABA agonist. In the short-term, these changes in cellular excitability can disrupt glutamatergic paired-pulse plasticity, enhance presynaptic GABA release, and suppress formation of LTP [135]. Chronic exposure to alcohol can result in long-term changes in expression of genes important for memory formation, as well as protein levels that facilitate the neuroanatomical correlates of learning, including neurotrophic factors. 

Numerous factors contribute to the beneficial effects of exercise on measures of learning, memory, and neuroplasticity in both the healthy and the damaged brain. Changes to levels of growth factors such as brain-derived neurotrophic factor (BDNF) and vascular endothelial growth factor (VEGF), alterations to neurotransmitter and hormone signaling, as well as anti-apoptotic and antioxidant effects have all been suggested as potential mechanisms. In addition, increases in cerebral blood flow and microvasculature are reported following bouts of exercise in humans and animals. The following sections discuss how both developmental alcohol exposure and exercise affect various molecular and neuroanatomical measures that could ultimately influence behavior and cognition.

### 3.1. Alcohol and Exercise Effects on Neurotransmitters, Neuromodulators and Hormones

Alcohol exposure not only affects the neuronal circuitry of the brain, but it also alters the development and functioning of the neurotransmitter and neuromodulator systems. Alcohol is an NMDA antagonist and a GABA agonist. During the period of intensive brain growth, NMDA receptors undergo a period of hypersensitivity resulting in NMDA-R-containing neurons to become exceedingly more sensitive to excitotoxic effects of alcohol and other NMDA antagonists (reviewed in [136]. In addition, focus has been made on the cholinergic system and it’s interaction with alcohol. Alcohol exposure during development, both neonatally and during adolescence, reduces levels of choline acetyltransferase in the adult animal, resulting in decreased acetylcholine availability [137]. Decreased levels of cortical acetylcholine are also a result of long-term moderate alcohol exposure in adult rats [138]. Cholinergic dysfunction is a common associated with memory impairments evident in Alzheimer’s patients [138,139]. Therefore, it may be possible that some of the memory impairments evident in FASD patients are associated with the cholinergic dysfunction. Thus, much work has focused on the role of cholinergic supplementation to rats following prenatal alcohol exposure and revealed very promising results wherein administration of choline to alcohol-exposed pups mitigates the detrimental influence of developmental alcohol exposure in the hippocampus [107,140] and the PFC [107]. 

Additionally, alcohol intake by a pregnant rat dam inhibits serotonin synthesis and the expression of serotonin precursor tryptophan hydroxylase in the dorsal raphe nucleus of her offspring [141]. Further, work in adult zebrafish illustrates that embryonic exposure to low doses of ethanol reduced levels of both dopamine and serotonin when measured in adulthood [142]. Alcohol exposure during adolescence enhances serotonin (1A) receptors in the dorsal raphe nucleus and reduces cannabinoid receptors in both the striatum and the globus pallidus when measured in adulthood [143]. Altered dopamine and serotonin levels have been implicated in many psychopathologies and may play a similar role in FASD. Chronic administration of alcohol during adulthood revealed the role of β-endorphins in modulation the locomotor effects of alcohol and how β-endorphins contribute to the neuroadaptive changes associated with chronic use [144]. Electrophysiology work using mice brain slices revealed that chronic alcohol exposure enhances the synaptic plasticity of NMDA receptors in the ventral tegmental area [145]. This drug-induced neuroadaptation may lead to enhanced drug-associated memories, thus making the animal more susceptible to drug intake later in life. Overall, the data illustrate a significant impact of alcohol on the developing brain and indicate that future interventions may benefit from targeting neurotransmitter systems in order to alleviate the effects of alcohol. 

More recently, studies have examined the interaction between alcohol and hormones. In particular, differing activation of the hypothalamic-pituitary-adrenal axis (HPA) axis and stress responsiveness is seen in males and females. Recently, it has been shown that 17β-estradiol may provide a neuroprotective influence in relation to the negative impact of alcohol exposure on the developing HPA axis. Administration of 17β-estradiol on PD4 and 5, thirty minutes prior to alcohol administration in rat pups served to attenuate alcohol induced cerebellar-associated behavioral deficits as well as the increased levels of antioxidants and enhanced lipid peroxidation process, which themselves could indicate increased oxidative stress, when assessed on PD23 [146]. Further, two studies by Przybycien-Szymanska and colleagues [147,148] revealed a protective role of 17β-estradiol in mitigating the negative influence of a repeated binge-pattern alcohol exposure during adolescence in females. Specifically, in males and ovariectomized females, adolescent alcohol exposure increases the expression of corticotrophin-releasing hormone and vasopressin, while this effect is not evident in hormone-intact females. This result indicates the necessity of 17β-estradiol of females in order for habituation of the HPA axis to repeated binge-pattern alcohol exposure to occur. Further, male and female pups that received gestational alcohol exposure exhibit differential stress responsiveness and HPA activity. Interestingly, in females, alcohol exposure delayed sexual maturation and altered HPA activity compared to control females in the same stage of the estrous cycle. This study suggests an alcohol-induced change in HPA activity that is estrous phase-specific [149]. Together, these data implicate the importance of estrogen hormones as a potential neuroprotective component of response to developmental alcohol exposure. 

Aerobic exercise has been targeted as a strategy to mitigate the impact of alcohol exposure on the proper functioning of neurotransmitter and neuromodulator systems, given its therapeutic role in abating deregulations in said systems in models of brain disorders. In humans, older individuals show enhanced cognitive memory impairments, which exercise has recently been shown to reduce [150]. In experimental animals, rats exhibit an elevated fear response following stress; however, exercise has been shown to reduce overactivation of the serotonergic neurons within the dorsal raphe following stress, while mirroring a blunting of the fear response [151]. Further, through the use of a rat model of Parkinson’s disease (rats were infused with a low dose of 6-hydroxydopamine in order to create a partial lesion of dopamine neurons) exercise was shown to have a neuroprotective effect on dopamine neurons. Specifically, exercise decreased asymmetry in the use of left and right forelimbs to explore a novel environment as well as decreased asymmetry in the number of tyrosine hydroxylase-positive cells in the substantia nigra pars compacta and decreased dopamine cell loss in 6-OHDA-lesioned rats [152]. Further, rodents expressing ADHD symptoms (spontaneously hypertensive) exhibit increased locomotor activity in an open field as well as impaired spatial memory on a radial arm maze. Exercise exposure for 28 days improved learning and memory, reduced open field hyperactivity, and increased levels of dopamine synthesis in the striatum and substantia nigra [153]. Moreover, reduced levels of serotonin, metabolite 5-HIAA, dopamine, and noradrenaline were observed across several brain regions in female R6/1 mice (model of Huntington’s Disease). Physical activity modulated these deficits [154]. Finally, in the elderly, exercise is positively correlated with increased levels of salivary alpha-amylase, suggesting upregulation of the noradrenergic system [150]. As such, aerobic exercise has also been shown to be a potential therapy for neurocognitive and mood disorders through its action on neurotransmitter systems and may similarly provide therapy to FASD patients. 

Exercise may also exert a therapeutic role on the damaged brain through altered levels of hormones. In humans, physical activity has been shown to be an important intervention when combatting disorders such as major depressive disorder (MDD). Lawlor and Hopker’s [155] meta-analysis revealed that exercise was as effective as cognitive therapy, though the mechanisms of exercise’s mood-enhancing effects has not been fully elucidated. Anhedonia is a primary symptom of MDD, and researchers have tried to model this aspect of the disorder using the forced swim test and measuring the amount of time the animal spends immobile. Increased immobility signals anhedonia, as the animal is not putting as much effort into escape. Sigwalt and colleagues [156] found that swimming exercise normalized hippocampal BDNF and interleukin-10 levels, as well as increased testosterone levels, suggesting a role for these proteins in reversing the anhedonic aspect of depression. Furthermore, there appears to be a protective interaction between estrogen and exercise. Ovariectomized animals are less active than intact females and exhibit decreased BDNF mRNA levels in comparison to sedentary controls. However, estrogen replacement promotes voluntary activity and, resultantly, enhances BNDF mRNA levels. In fact, exercise plus long-term estrogen replacement increases BDNF protein levels above those of estrogen replacement alone [157]. Similarly, work by Erickson and colleagues [158] examined the interaction of exercise and hormone replacement therapy in postmenopausal women. The results indicated that the combination therapy not only improved performance in executive functioning but also spared gray matter. Interestingly, the results also indicated that higher fitness levels could offset the associated risks of a longer hormone replacement therapy. These data suggest a dual treatment of exercise plus estrogen may be optimal to combat decreased BDNF levels evident in FASD patients. 

### 3.2. Neurotrophic Factors

Neurotrophic growth factors are a family of proteins important for the differentiation, maturation and survival of neurons in the brain, both during initial development and during adulthood [159]. In particular, BDNF has been shown to be particularly important for cellular migration and maturation in the adult brain [160]. In addition, BDNF depletion in mice reduces dendritic complexity of hippocampal neurons. According to Kaufmann and Moser [161], “dendritic abnormalities are the most consistent anatomical correlates of mental retardation”, suggesting that changes to neuronal maturation and complexity related to altered BDNF levels could contribute to behavioral deficits similar to those seen in individuals with FASD. In addition, alcohol exposure in the adult brain blocks the enhancement of NMDA receptor function that is caused by BDNF expression [162]. NMDA receptors are critical for the induction of LTP, suggesting another mechanism through which alcohol exposure could enact long-term effects on cognition and memory.

Developmental alcohol exposure can have a long-lasting impact on BDNF levels in the brain, perhaps contributing to alcohol-related deficits in learning and cognition (summarized in Table 2). However, the effect of alcohol exposure on BDNF seems to be brain region-specific and could be dependent on dose and timing of the exposure. Early work by Heaton and colleagues [88] showed that levels of BDNF, nerve growth factor (NGF), and neurotrophin-3 (NT-3) in rats were altered more quickly following postnatal alcohol exposure as compared to following prenatal exposure and levels differed based on region of interest. In fact, BDNF levels were increased only following exposure on PD 4-10. This increase was seen directly after the completion of the alcohol exposure, meaning that it could be an immediate neuroprotective response of the brain to the presence of alcohol. Similar increases were shown in the cerebellum immediately following alcohol exposure on PD 4 [89]. As the time following the exposure increased, BDNF levels decreased below the control baseline. Mirroring these results, Light, Ge, and Belcher [91] reported decreased cerebellar BDNF and TrkB receptor mRNA 24 h after PD 2 and 3 alcohol exposure. These alterations to cerebellar BDNF levels could contribute to the decreased Purkinje cell and synapse number and motor task learning deficits reported following postnatal alcohol exposure.

More recently, Fattori, Kocayashi, and Tsuji [87] reported that cortical BDNF levels were decreased following postnatal alcohol exposure. In addition, levels of MAPK, a protein involved in an important downstream signaling pathway that affects gene expression related to learning and memory, were decreased. The MAPK pathway is one possible route alcohol exposure might contribute to behavioral deficits observed following development alcohol exposure. BDNF has also been suggested to play a role in alcohol-related depressive-like effects in mice [66]. Perinatally-exposed mice displayed increased learned helplessness and anhedonia as measured by immobility during a forced swim test. While hippocampal BDNF levels were not affected, medial PFC levels were significantly reduced in these mice, again supporting the brain region-specific effects of alcohol on BDNF. Central administration of BDNF also reduces anxiety-like behaviors and normalizes aberrant sexual preference and social behaviors in prenatally alcohol exposed male mice born to dams also exposed to stress [163], suggesting that altered BDNF levels might affect these types of behaviors.

However, not all experiments that look at the long-term impact of developmental alcohol exposure find that alcohol decreases BDNF expression—the relationship between alcohol and BDNF is not clear cut. For example, Boehme and colleagues [52] found no effect of perinatal alcohol exposure on BDNF protein levels on PD 60. Conversely, other studies have found that developmental alcohol exposure increases brain BDNF levels in adult and aged mice [86,92]. Acute adolescent alcohol exposure on PD 23 also increases BDNF levels within the hippocampus [90]. Thus, no consensus has been reached regarding developmental alcohol exposure effects BDNF and how this protein is related to alcohol-related behavioral deficits. More research is needed to understand the complex relationship between alcohol and neurotrophic factor expression, particularly in the adolescent brain. However, it is clear that alcohol exposure does alter BDNF expression and that BDNF is an important factor in neuronal maturation and signaling pathways related to learning and memory.

Aerobic exercise has been shown to robustly enhance BDNF expression throughout the brain (Table 2). In addition, activity-dependent enhancement of BDNF expression has been suggested as a mechanism through which exercise improves learning and memory [164]. Notably, BDNF promotes neuronal differentiation, maturation, and survival. In addition, increases in BDNF enhance LTP, affect gene transcription related to learning and memory, and contribute to increased dendritic complexity [1,165]. 

Rasmussen and colleagues [95] showed that intense rowing exercise in human adults increases plasma BDNF. Even 20 min of vigorous exercise enhanced BDNF expression in men and women, though there did seem to be a more pronounced effect in women [96]. Additionally, enhanced BDNF expression has been demonstrated in the mouse hippocampus and cortex following two hours of treadmill exercise [95]. Griffin and colleagues [75] also showed that one week of treadmill exercise significantly enhances BDNF levels throughout all subfields of the hippocampus and perirhinal cortex. Further, this study reported spatial and nonspatial learning improvements in exercised rats. Ding, Ying and Gómez-Pinilla [93] demonstrated that one week of aerobic exercise affects the BDNF signaling pathway, increasing both the precursor and mature forms of the BDNF protein.

In addition to having positive effects in the healthy brain, exercise also can increase BDNF expression in models of brain damage or aging. Marlatt and colleagues [77] reported that female mice given access to running wheels throughout middle age showed increased BDNF levels which correlated with improved spatial memory and increased hippocampal adult neurogenesis at 15 months of age. In addition, three months of exercise increased BDNF in a transgenic mouse model of Alzheimer’s disease [97]. The transgenic mice, which were 24 months of age at exercise onset, also exhibited reduced neuronal death compared to control mice, possibly in part due to the role of BDNF in cell survival. Also, exercise may be a potential therapy in rodent stroke models. Ke and colleagues [166] demonstrated that rats that experienced treadmill exercise following induction of an ischemic stroke had enhanced hippocampal BDNF concentrations, as well as improved recovery of motor function. A blockade of BDNF mRNA production reduces the therapeutic benefit of a skilled reaching task following an ischemic lesion in rats [167], suggesting that BDNF has a necessary role in relearning of a motor task, perhaps through effects on post-damage reactive neurogenesis or strengthening pre-existing connections. 

Finally, one particular study highlights an important role for exercise-induced BDNF in learning. Mice exposed to exercise for one week prior to exposure to the Morris Water Maze task displayed enhanced spatial memory and BDNF levels in the hippocampus [79]. When hippocampal TrkB receptors were blocked during the pre-testing exercise period, no improvement in performance was seen, suggesting that BDNF is necessary for exercise-induced memory enhancements. Overall, current evidence implicates BDNF as a prime candidate for involvement in mechanism underlying exercise-induced neuroplasticity.

### 3.3. Microvasculature

Aside from their role in maturation and survival of cells comprising the nervous tissue, growth factors in the brain can affect the extension and complexity of microvasculature that supplies necessary nutrients and oxygen to cells. For example, vascular endothelial growth factor (VEGF) is critical for angiogenesis during development and adulthood, and can affect the permeability of the blood-brain barrier [168]. Changes to microvasculature could affect the function and survival of neurons due to a lack of essential nutrients or hypoxia, perhaps ultimately contributing to behavioral or cognitive deficits.

Very little research to-date has looked at the effect of developmental alcohol exposure on VEGF levels per se, but current evidence does support alterations to cerebral blood flow caused by alcohol (Table 3). For example, decreased blood flow to the brain for at least 24 h was observed in mice fetuses exposed to a single or repeated administration of alcohol [120]. This study furthers previous work done by Jones, Leichter and Lee [122] that reported reduced blood flow to the placenta in rat models of prenatal alcohol exposure, and suggests that changes to blood flow during development could contribute to decreased birth weight and brain volume in the offspring. Conversely, Parnell and colleagues [169] found increased cerebellar blood flow in a sheep model of FASD that correlated with reduced Purkinje cell numbers, suggesting alcohol-induced alterations to cerebral blood flow might differ based on brain region and dose.

Alterations to cerebral blood flow can be long-lasting. In humans, children with FAS had hypoperfusion of blood to the left hemisphere when measured by single-photon emission computed tomography (SPECT) [123]. Similarly, Bhatara and colleagues [121] reported that cerebral blood flow to the temporal lobe (and thus, possibly the hippocampal formation) was decreased by 25% in patients with FAS. Changes to cerebral blood circulation and microvasculature have not been well studied in animal models, but there is some evidence that prenatal alcohol exposure has a long-lasting impact of the efficiency of cerebral arteries. Mayhan [170] reported that rats exposed to alcohol during adulthood had reduced vasodilation following administration in response to drugs that normally increase blood vessel diameter. Similarly, Gleason and colleagues [171] reported that young sheep prenatally exposed to alcohol had impaired attenuation of blood vessel size in response to hypoxia. Taken together, these results suggest alterations to vasculature that could have negative functional implications later in life, though further research should be done to fully explore these effects.

Recently, VEGF has also been suggested to have an important role in neuroplasticity, particularly adult neurogenesis [172]. Developmental alcohol exposure impairs adult neurogenesis [53,54,116], possibly due in part to alterations in growth factors such as VEGF. Few studies have investigated how alcohol exposure affects VEGF. One study by Ceccanti and colleagues [86] reported that prenatal alcohol exposure significantly increased VEGF expression in the cortex and hippocampus when measured during adulthood. Similarly, in adult rats, chronic alcohol drinking increased VEGF expression, contributing to the protective effect of alcohol consumption during cerebrovascular events and ischemia [173]. However, in their paper, Ceccanti and colleagues [86] suggest that increased VEGF might also contribute to tumor proliferation and angiogenesis, leading to increased health risk later in life.

Aerobic exercise has profound influences on cerebral blood flow, microvasculature and VEGF expression (Table 2 and Table 3). In humans, aerobic exercise increased frontal cortex blood flow and oxygen delivery with more intense exercise creating a greater enhancement [126]. Chronic exercise could induce long-term changes to the blood flow to the frontal cortex, perhaps affecting performance on executive functioning tasks. Sato and colleagues [124] also reported increased blood flow through the carotid and vertebral arteries in an intensity-graded manner in adults involved in graded cycling exercise. Exercise also has beneficial effects at the cellular level, as shown by Latimer and colleagues [99]. In their study, six weeks of wheel running exposure in middle-aged female mice significantly enhanced VEGF expression and reduced diastolic blood pressure in these animals. Further, electron microscopy revealed that the endothelial cells of the middle cerebral artery were much larger and smoother in appearance, similar to endothelial cells in young animals, demonstrating that exercise had a positive effect on the integrity of arteries involved in the blood-brain barrier. 

Recent studies have also investigated the time course of the beneficial effects of exercise microvasculature and found that the system is quite dynamic. Research involving macaque monkeys showed that five months of one hour per day treadmill exercise increased vascular density in the motor cortex and needed fewer trials to reach criterion on a spatial learning task [78]. However, chronic access to physical exercise was needed for these benefits, as the effects were reversed in monkeys kept sedentary for three months following exercise access. The remarkable plasticity of the brain in response to changes in physical exercise demand were further illustrated by Van der Borght and colleagues [125]: when mice were given access to one, three or ten days of access to a running wheel, blood vessel density was significantly increased after only three days. Levels of new cell proliferation in the hippocampus were also increased by the 10 day mark in these mice. However, both of these measures returned to baseline after only 24 h following removal of the running wheel. While more chronic exposures to exercise might induce longer-lasting changes, a continued exposure to the exercise paradigm seems to be most conducive to producing substantial changes in microvasculature and blood flow.

As mentioned previously, the angiogenic protein VEGF has been suggested to be important for neuroplasticity [91] and upregulation of this protein by physical activity could contribute to exercise-induced enhancement in plasticity. In a recent study, chronically stressed mice display decreased hippocampal blood vessel density and adult neurogenesis, alongside increased anhedonic behavior as measured in the forced swim task [98]. Stressed animals that were exposed to one hour of treadmill exercise per day for two weeks showed significantly normalized behavior and adult neurogenesis. However, the beneficial effect of exercise was blocked when the mice were also administered a VEGF Flk-1 receptor antagonist. These results suggest that the Flk-1 receptor might be important for the positive effects of exercise on neuroplasticity and make this receptor an important target of future research in models of developmental alcohol exposure.

### 3.4. Oxidative Stress

Another potential mechanism of alcohol’s effects on cytotoxicity and neuroplasticity is increased oxidative stress, which can damage cells and DNA through the production of peroxidases and other free radical molecules (Table 2). Ramachandran and colleagues [101] (Table 2) described that acute prenatal alcohol exposure in fetal rat cortical neurons increased levels of reactive oxygen species and was followed by apoptosis. Pretreatment with an antioxidant prevented the cortical apoptosis, suggesting that the rise in oxidative stress was directly linked to the increased cell death. Sun and colleagues [174] reported similar findings when acute alcohol administration increased levels of the oxidative stress marker lipid peroxidase *in vitro*. This finding was supported by *in vivo* observations that postnatal alcohol exposure in rats increases in lipid peroxidase levels throughout the brain, as late as 12 weeks after exposure [100]. Further, recent work by Johnsen-Soriano and colleagues [175] in adult alcohol-treated animals found significantly increased levels of lipid peroxidase, as well as impaired hippocampal LTP. Treatment with an antioxidant compound reversed the deficits in LTP, as well as decreased lipid peroxidase biomarkers. Finally, perinatal alcohol exposure also resulted in long-lasting increases in lipid peroxidase and protein oxidation levels within the hippocampus and cerebellum [62]. These changes were present alongside increased anxiety- and depressive-like behaviors in the mice. 

Notably, Brocardo and colleagues [62] reported that access to wheel running reversed the enhanced levels of lipid peroxidase, as well as increased glutathione markers, an antioxidant. Further, wheel running ameliorated behavioral deficits in the male animals. While the effect of wheel running on oxidative stress markers in alcohol-exposed animals has not been fully explored, evidence shows that physical activity reduces levels of oxidative stress in healthy brains, as well as other models of disease. In rodents, exercise reduces levels of reactive oxygen species [176,177] and oxidative protein damage [178], and increases levels of some endogenous antioxidants [176]. 

Tissue and DNA damage cause by free radical accumulation has also been suggested as an underlying cause of the aging process [179,180]. Thus, some research has focused on reducing oxidative stress markers in aged animals through exercise. Marosi and colleagues [181] gave middle-aged rats 15 weeks of wheel running access and investigated the effects on oxidative stress markers in the hippocampus. In the aged rats, exercise reduced levels of reactive oxygen species and increased endogenous antioxidants, showing that exercise is just as effective in older animals as young animals. However, this study did not have a younger control group to investigate if these biomarkers were altered in the aged animals to start. A later study by Cui and colleagues [182] demonstrated that sedentary aged rats had increased DNA oxidation and lipid peroxidase levels compared to young rats, and that these increases could be reversed to the level of the young control animals through lifelong (94 weeks) access to a running wheel. Three months of exercise late in life was also shown to have beneficial effects, though not as profound as effects seen in the lifelong exercise animals. Finally, Navarro and colleagues [183] showed that even relatively small amounts of exercise throughout the lifetime can lengthen the lifespan in mice, as well as reduce oxidative stress markers well into adulthood.

### 3.5. Cytotoxicity/Apoptosis

Apoptosis refers to programmed cell death and it is a critical natural phenomenon present in proper brain development. During development an overproduction of cells occurs and, as such, cells compete to migrate and incorporate into the developing neuronal networks. This competition leads to a survival of the “fittest” neurons and apoptosis serves as a natural mechanism to eliminate the unneeded neurons. Alcohol exposure on the developing brain has consistently been proven to increase levels of apoptosis (Table 2). Previous work examined the mechanisms of alcohol as an NMDA antagonist and GABA agonist in apoptotic activation. Specifically, Olney and colleagues [102] argued and successfully demonstrated that alcohol triggers apoptotic neurodegeneration by a dual mechanism-blockade of NMDA glutamate receptors and excessive activation of GABA_A_ receptors and that certain brain regions are more susceptible than others as this is dependent on whether physiological apoptosis is ongoing in a brain region. 

Alcohol-induced activation of caspase-3, a cysteine protease enzyme, paralleled that of alcohol-induced apoptotic neurodegeneration in both the hippocampus and the cortex [102,103]. Furthermore, administration of alcohol during the third trimester equivalent (PD7-9; 5 g/kg) increased neuronal apoptosis and levels of NF-κβ and caspase-3 in both the cerebral cortex and the hippocampus when assessed on PD28 [105]. Additionally, PD7 alcohol exposure (5 g/kg) induced upregulation of Bax, release of mitochondrial cytochrome-*c* into the cytosol, activation of caspase-3 and cleavage of poly (ADP-ribose) polymerase (PARP-1), all of which promote apoptosis [106]. Together, these data implicate caspase-3 as a modulator in alcohol-induced apoptosis. 

Another mechanism through which alcohol may induce widespread apoptotic neurodegeneration throughout the forebrain during development is activation of ceramides, a family of lipid molecules that serve as a signaling molecule for many developmental factors, including apoptosis. Examination both 2 h and 24 h following one acute exposure of alcohol in PD 7 mice indicated the apoptotic-inducing ramifications of developmental alcohol exposure to the developing brain. In addition, alongside the alcohol-induced apoptotic neurodegeneration in the brain was elevated levels of GM2 ganglioside. This elevation was small but significant two hours following alcohol exposure and massive by 24 h [104]. Gangliosides are involved in apoptotic pathways given that they are synthesized from ceramide by sequential glycosylation. Ceramide synthesis is critical for alcohol-induced apoptotic pathway [184]. Further, PD 7 alcohol exposure induced sphingosine kinase 2 activation and increased the brain level of sphingosine 1-phosphate (S1P), a ceramide metabolite, transiently 2- to 4 h after exposure, followed by caspase-3 activation that peaked around 8 h after exposure [185]. Together, these data implicate a relationship between ceramide synthesis and alcohol-induced apoptotic neurodegeneration. 

Further, the toxicity of alcohol of the developing brain affects other factors in cell development. VanDenmark and colleagues [186] treated prenatal rat hippocampal pyramidal neurons with alcohol in the presence or absence of cholinergic agonist carbachol for 24 h. Alcohol-treatment for 24 h significantly inhibited carbachol-induced increase in intracellular calcium and carbachol-induced axonal growth. Also, alcohol inhibited carbachol-induced neurite outgrowth by inhibiting PKC and ERK1/2 activation. Prenatal exposure of C57Bl/6 mice to alcohol elicited an increase in TUNEL-positive cells relative to control animals in all hippocampal regions as well as reduction in both neuronal and glial cell densities, suggesting both cell types are sensitive to the neurotoxic effects of alcohol. Overall the data demonstrate multiple stages at which alcohol can affect, and ultimately increase level of apoptosis.

Aerobic exercise has consistently been proven to decrease levels of apoptosis in the damaged brain (Table 2). Specifically, it may do so by enhancing levels of caspase-3, one of the key factors in the apoptotic pathway. One study, in humans, examined the impact of resistance exercise on serum p53, caspase-9, and caspase-3, markers of apoptosis. Results of this study revealed immediately post exercise training, control subjects had increased levels of p53, caspase-9, and caspase-3 compared to those who underwent resistance training. This data suggests, prior training altered apoptosis biomarkers, specifically caspase-3 [187]. Natural aging increases apoptosis levels in the brain. Rodent work demonstrates that treadmill exercise decreases apoptosis levels as well as DNA fragmentation and caspase-3 expression in the hippocampus in aged animals when compared to aged animals that did not exercise [188]. Furthermore, Alzheimer’s disease is associated with increased levels of apoptosis. Transgenic mice that serve as a model for Alzheimer’s disease underwent treadmill exercise for twelve weeks starting at 24 months of age. The result of this exercise intervention was a significant reduction in the expression of Aβ-42, Cox-2, and caspase-3 in the hippocampus as well as altered phosphorylation levels [97]. Further, enhanced apoptosis is evident in the brain of animals born to hyperthermiac mothers. However, if the mother exercised following the hyperthermia experience apoptosis were downregulated as are levels of caspase-9, -7, and -3, implicating the importance of maternal exercise in inhibiting apoptotic cell death in embryos against hyperthermic exposure during pregnancy [189]. Overall, these data indicate the powerful therapeutic advantage of exercise in decreasing levels of capase-3, which is associated with decreased levels of apoptosis. 

More recently, work has begun investigating if reduced ceramide levels could also decrease apoptosis. One study by Snigdha and colleagues [190] examined the impact of various interventions (behavioral enrichment and antioxidant diet) combined or separately on caspase activation and ceramide accumulation in aged beagles. Behavioral enrichment consisted of pair housing animals and providing them with physical exercise and cognitive enrichment. This enrichment reduced levels of both activated caspase-3 and ceramide [190]. Little work has examined the impact of physical exercise alone on ceramide levels in the brain. More recently, analysis of lipid content in the hypothalamus following high-fat feeding in rodents demonstrated an increased lipid content, including ceramide levels, that was not reduced following six weeks of exercise training [191]. Comparatively, muscular ceramide levels are affected by exercise as rats exposed to a high fat diet exhibit higher plasma lipid profiles when compared to those of exercise alone or high-fat diet animals that exercised [192]. Together, these data illustrate the powerful influence of exercise in reducing abnormal levels of apoptosis in the damaged brain. Interestingly, it achieves this effect by targeting many of the same factors that are altered by developmental alcohol exposure. These data support that exercise may serve as a suitable intervention to mitigate the alcohol-induced apoptosis evident in the developing brain. 

## 4. Exercise as a Therapeutic Intervention to Restore Brain Structure and Function after Developmental Insult

The ability of exercise to restore proper brain function following developmental insult has been illustrated in the literature. Rodent models mimicking early life stress, through maternal separation, demonstrate the long-term structural and functional alterations. For example, in the hippocampus, expression levels of many proteins involved in neuronal structure, metabolism, signaling, anti-oxidative stress and neurotransmission occur due to maternal separation [193]. Additionally, these animals exhibit increased anxiety-like behaviors and significant reductions in hippocampal glucocorticoid receptors as well as BDNF and serotonin receptor mRNA [194]. When these animals exercise voluntarily during adolescence, this activity normalizes the early life stress-induced hippocampal deficits [193,194]. Exercise exposure also improves forelimb stimulation; the percentage and magnitude of responding cells to said forelimb stimulations in adult rats subjected to thoracic transections as neonates [195]. Further, exposure of PD 7 rat pups to a unilateral hypoxic-ischemic insult produced long-term deficits in spatial learning when tested 16 weeks later. Interestingly, exposure to a rehabilitative training for ten weeks prior to testing was able to rescue deficits in female rats but not in male rats [196]. These data suggest gender may be a critical factor to consider when developing cognitive rehabilitation programs.

Based on exercise’s robust effects on both the health and damaged brain, recent research related to developmental alcohol exposure has explored the validity of exercise as potential therapeutic treatment for brain-related deficits. It is clear from the literature presented in the previous section that aerobic exercise affects many of the same systems that are negatively impacted by alcohol (summarized in Table 1). Understanding how physical activity can be used as a low-cost, easily accessible behavioral intervention for children with FASD or adolescents who drink systematically is an important next step in improving current treatments for these individuals. Below, we describe the research that has been done to-date on the impact of exercise on alcohol-related deficits.

### 4.1. Therapeutic Role of Exercise Following Developmental Alcohol Exposure

Developmental alcohol exposure can inflict devastating, long-lasting behavioral and neuroanatomical impairments on the child. While deficits caused by alcohol exposure are completely preventable, many women continue to drink while pregnant and teenagers are experimenting with alcohol at younger ages than ever before. Thus, it is important to investigate therapeutic interventions that can ameliorate some of the damage caused by alcohol exposure during development. Aerobic exercise has been shown to have positive effects on the damaged brain, including deficits caused by aging, stress, and drug addiction (reviewed in [1]). Animal research investigating the effects of exercise in models of FASD is rather limited, but the research available promotes the theory that exercise can help ameliorate alcohol-induced neuroanatomical and behavioral impairments. To date, virtually no work has been done examining the possible benefits of pure aerobic exercise on children with FASD, but hopefully the growing literature on the positive results in animal models will encourage clinicians to view exercise as a potential new therapeutic avenue. This section will briefly describe current evidence supporting a significant and beneficial effect of exercise following developmental alcohol exposure in animals. 

### 4.2. Exercise Effects in FASD Models

As described in the previous section, developmental alcohol exposure can disrupt balance and acquisition of fine motor tasks through damage to the cerebellum. Most of the work focusing on the effects of behavioral interventions on the cerebellum has used complex motor tasks that combine training on both fine motor skills and balance with increased locomotor activity. Work from the Greenough lab group has explored how running *versus* training on a complex motor task can enhance cerebellar plasticity and performance on a balance task. Specifically, exposure to 4.5 g/kg/day of alcohol on PD 4–9 reduced Purkinje and granule cell number, and also impaired performance on a complex motor task [69]. Rats were trained for 10 days on an acrobatic obstacle course; alcohol-exposed rats initially took longer to traverse the obstacles but by the end of training they performed as well as control animals. When compared to inactive alcohol-exposed rats, alcohol-exposed rats trained on the acrobatic task had significantly more parallel fiber synapses per Purkinje cell. Further work also described that 20 days of training increased volume of the paramedian lobule in alcohol-exposed rats [70]. Complex motor training, but not pure running, also has been shown to improve performance on other motor tasks, such as balancing on a rotating rod [36]. Recently, Brocardo and colleagues [62] reported that aerobic exercise was able to reverse increased levels of oxidative stress in the cerebellum of alcohol-exposed rats following alcohol exposure during all three trimester-equivalents. Overall, these data suggest that motor training can enhance synaptogenesis in the cerebellum that might be contributing to improving motor deficits seen in children with FASD. 

In rodents, exercise has also been shown to enhance performance on hippocampal-associated tasks of spatial memory. Alcohol exposed (either prenatal or postnatal exposure) rats have been reported to display impaired spatial memory on the Morris Water Maze task [40,41]. However, extended access to a running wheel shortened the latency to the platform in the alcohol-exposed group to a level indistinguishable from controls in all cases. In addition, aerobic exercise can reverse alcohol-related impairments on neurophysiological measures of synaptic plasticity. Exercise can enhance LTP in the perforant pathway in prenatally exposed animals [40], as well as increase hippocampal expression of immediate early gene c-Fos, which is a marker of neuronal activation [197]. 

Notably, exercise interventions models of FASD with prenatal and/or postnatal exposure have been shown to increase new cell proliferation in the dentate gyrus [52,198,199]. Conflicting results have been found for exercise’s effect on new cell survival in alcohol-exposed animals; Helfer and colleagues [199] found that 30 days of social housing following 12 days of voluntary exercise was not sufficient to enhance cell survival in neonatally alcohol-exposed animals, while cell survival was increased in control animals. However, Boehme and colleagues [52] have reported a robust benefit in cell survival 28 days after 12 days of wheel running in animals exposed through all three trimester-equivalents. Reasons for these conflicting results could be the sex or genetic strain of the animals used, as well as differing developmental time points for wheel running access (early *versus* late adolescence) and tissue analysis. 

Perinatally alcohol-exposed animals also show increased anxiety- and depression-like symptoms as measured on an elevated plus maze, an open field test, and a forced swim test [62]. Twelve days of wheel running showed mixed impact on these symptoms, and some of the effects differed based on sex on the animal. Male alcohol-exposed rats showed anti-anhedonic effects of wheel running in the forced swim test as evidenced by less time spent immobilized in the water; conversely, female rats did not show this benefit. No positive effect was shown in the open field or elevated plus maze. Thomas and colleagues [41] also reported increased locomotion in the open field test in neonatally alcohol-exposed rats; this overactivity was ameliorated by 30 days of wheel running during adolescence. Overall, the above studies support that exercise is a potential behavioral therapy for individuals with FASD, particularly if the intervention given is in childhood or adolescence.

### 4.3. Exercise Effects in Models of Adolescent Alcohol Exposure

Adolescence is a time period in which society encourages children to become more involved with social groups and sports activities. In particular, a higher frequency of moderate or physical exercise is associated with improved psychological well-being for adolescents [200]. Further, adolescent athletes report better sleeping patterns as well as less tiredness and increased concentration during the day [201]. Exercise has been shown to enhance brain plasticity in both children and adults, yet the data is lacking on whether benefits in brain plasticity are evident in humans during adolescence—a highly plastic developmental time window. Recently, Herting and Nagel [76] examined the influence of exercise on hippocampal structure and function in adolescent males. The results of this study indicate that in adolescent males, aerobic fitness predicted larger hippocampal volume and was associated with better visuospatial learning. 

In adolescent rodents, aerobic exercise appears to boost both behavioral and anatomical measures of plasticity. Behaviorally, the impact of exercise during adolescence appears to be different than the impact of exercise during adulthood [202]. Specifically, adolescents exposed to four weeks of voluntary exercise performed similarly to control animals on a novel object recognition task immediately following exercise and such outcome persisted for two or four weeks after the exercise treatment. However, while adult rats exposed to the same exercise treatment showed improved novel object recognition performance immediately following exercise, no effect of exercise was evident two or four weeks following its completion. In addition, exercise improved spatial learning and memory and enhanced capacity to evoke spatial memories in later stages. Further, it produced an increase in mossy fiber density and hippocampal expression of BDNF and TrKb as well [94]. Exposure to voluntary exercise resulted in a significant increase of protein level in the hippocampal formation and PV-immunoreactive neurons in CA1 and CA2/CA3 regions [110]. Additionally, exercise enhanced BDNF levels in the perirhinal cortex and hippocampus in adolescents and adults immediately following exercise, yet the long-term impact of exercise on BDNF levels was only evident in the adolescent rats. Interestingly, voluntary exercise has been shown to enhance LTP in the adolescent male, but not the adolescent female, dentate gyrus [85], suggesting a sex difference. Overall, the data imply adolescents may benefit greatly from exercise exposure and that it may serve as a therapeutic strategy to enhance brain plasticity in the damaged brain. 

In adolescents, a correlation between substance abuse and decreased physical activity occurs. A longitudinal study examining substance abuse and exercise participation from 1991 to 2009 indicates that higher levels of exercise were associated with lower levels of alcohol use and exercise helped suppress the relationship between team participation and alcohol use [203]. In addition, Buscemi and colleagues [204] recently exploring the impact of drinking and exercise in college students. A positive relationship between exercise and alcohol use was seen among men and students in the Greek system yet exercise did not appear to serve as a protective factor for college students. Still, the alcohol field should explore this relationship further and determine whether the plastic benefits of exercise could combat the detrimental influence of alcohol in the adolescent brain, as it appears to in the developmental brain. 

## 5. Exercise in Other Interventions

Exercise alone is rightfully considered to be a powerful enhancer of functional and structural plasticity, with effects that include, but are not limited to, improved spatial memory, upregulation of neurotrophic factors, increased cell proliferation [205,206,207], and survival in neurogenic brain regions [208,209,210,211]. Environmental complexity (EC) (or enrichment) is another powerful intervention: its effects were first demonstrated on a neuroanatomical level as significant increases in cortical weight and thickness, dendritic branching and synaptogenesis resulted from EC exposure [212,213,214]. These original experiments involving EC employed large animal cages (15–20 times larger than a standard rat cage) with toys being replaced at a regular interval, allowing for significant levels of exercise through exploration, social interaction, and novelty.

In some later studies, running wheels were added to the EC cages suggesting that it would provide more opportunity for experimental animals to exercise. Resultantly, the specificity of each EC component’s influence on plasticity emerged, leading to the question: is there a dissociation in the effects of EC and wheel running on brain plasticity? Recently several papers have emerged arguing that voluntary exercise in running wheels is indeed the most important and effective component of the EC paradigm [215,216]. The outcomes of these studies demonstrate the importance of running above other sensory stimulation in EC for adult neurogenesis and hippocampus-associated learning. However, other studies repeatedly demonstrated that learning, not mere exercise, is important for brain plasticity enhancement. For example, complex motor task learning (“acrobat training”) resulted in significant increase in cerebellar and motor cortical synaptogenesis while treadmill running failed to achieve the same effect [217,218]. In our studies of the effects of exercise on adult hippocampal neurogenesis in an animal model of binge drinking during the third trimester equivalent, wheel running alone was not sufficient to increase survival of new adult-generated neurons [117]. However, when animals experienced voluntary exercise for twelve days followed by thirty days in EC, adult neurogenesis [119] and hippocampus-associated behavior were rescued in alcohol-exposed animals [82]. These findings suggest that EC and voluntary exercise modulate different, possibly complementary, brain plasticity mechanisms in the alcohol-damaged brain.

## 6. Conclusions

Exercise is known to improve brain resilience to developmental trauma by promoting neuronal and vascular plasticity and is one of the prime candidates for FASD-related behavioral rehabilitation. As was discussed in this review, a complex picture of involvement of many cellular and subcellular processes in the mechanisms orchestrating the effect of exercise has begun to emerge. Mechanisms that we focused on in this review are alterations to signaling proteins, structural changes to microvasculature, enhancements in cerebral blood flow bringing vital nutrients to cells, and reductions in apoptotic proteins. In addition, precise spatial and temporal expression of neurotrophic factors, such as BDNF, is essential for a favorable outcome of exercise on the brain. The literature reviewed here support exercise as a very promising experimental avenue to reverse anatomical and behavioral abnormalities.

Exposure to alcohol during development (either pre/neonatal or adolescent) has significant negative consequences on the brain such as, but not limited to, increased apoptosis, decreased neurogenesis and angiogenesis, and altered expression of neurotrophic factors. Developmental alcohol exposure has both a monetary and emotional cost for the individual and for society. Despite increased awareness, rates of FASD remain stable. Thus, it is imperative that new intervention strategies be developed. More recently, the field has begun to explore potential benefits of physical activity on the damaged brain. Already exercise has been shown to ameliorate developmental alcohol-induced deficits such as decreased LTP, impaired spatial memory, hyperactivity, and lowered levels of hippocampal adult neurogenesis. While the available data is far from being exhaustive and complete, it nevertheless provides evidence in support of exercise as a mean of therapeutic intervention.

In sum, during the past two decades, the beneficial effects of exercise on the alcohol-damaged brain in animal models of developmental exposure received more and more convincing support. Aerobic exercise is a valuable therapeutic model as it could translate easily to a clinical setting. In humans, using exercise as a postnatal intervention would be accessible to people from many different socioeconomic statuses and life situations. Further, implementation of this therapy would be simple and cost effective. Finally, there is a considerable amount of literature in support of the robust beneficial effect of exercise. It is important that future work investigate functional and structural correlates of this potentially powerful therapeutic intervention on the alcohol-damaged brain and behavior. 
